# Supplementary material for: Cognitive aptitude, peers, and trajectories of marijuana use from adolescence through young adulthood
Source: PLoS One. 2019 Oct 25;14(10):e0223152. doi: 10.1371/journal.pone.0223152 (PMC6814275; doi:10.1371/journal.pone.0223152)
Supplement: S1 Table — (DOCX) [file pone.0223152.s001.docx]

**Supplemental Table S1. Cognitive Aptitude and Heterogeneous Trajectories of Marijuana Use with Inclusion of Peer Drug Use: Full Models (n=3,903)**

|  | | | | | | | | |
| --- | --- | --- | --- | --- | --- | --- | --- | --- |
|  | Occasional Dabblers | | Consistent Users | | Persistent Heavy Users | | Early Heavy Quitters | |
|  | Model 2 | Model 3 | Model 2 | Model 3 | Model 2 | Model 3 | Model 2 | Model 3 |
|  | Relative Risk  (95% CI) | Relative Risk  (95% CI) | Relative Risk  (95% CI) | Relative Risk  (95% CI) | Relative Risk  (95% CI) | Relative Risk  (95% CI) | Relative Risk  (95% CI) | Relative Risk  (95% CI) |
| Cognition | 1.048*  (1.007-1.090) | 1.054**  (1.013-1.097) | 1.126***  (1.066-1.189) | 1.134***  (1.074-1.199) | 1.030  (0.972-1.091) | 1.052  (0.992-1.115) | 0.917**  (0.871-0.965) | 0.937*  (0.889-0.987) |
| Peers Drug Use |  |  |  |  |  |  |  |  |
| About 25% |  | 1.410**  (1.098-1.810) |  | 1.806**  (1.290-2.528) |  | 1.756**  (1.169-2.636) |  | 1.454*  (1.026-2.061) |
| About half (50%) |  | 1.934***  (1.457-2.569) |  | 1.938**  (1.279-2.937) |  | 3.471***  (2.273-5.300) |  | 2.130***  (1.445-3.139) |
| About 75% |  | 1.918***  (1.347-2.732) |  | 2.537***  (1.572-4.096) |  | 5.460***  (3.430-8.691) |  | 3.752***  (2.472-5.696) |
| Almost all (more than 90%) |  | 1.626*  (1.048-2.524) |  | 1.928*  (1.016-3.660) |  | 4.274***  (2.403-7.605) |  | 4.704***  (3.014-7.344) |
| Depression | 1.093***  (1.052-1.135) | 1.092***  (1.051-1.135) | 1.104***  (1.048-1.163) | 1.103***  (1.047-1.163) | 1.109***  (1.049-1.172) | 1.104**  (1.043-1.168) | 1.096***  (1.044-1.150) | 1.088**  (1.037-1.142) |
| Age at baseline |  |  |  |  |  |  |  |  |
| 13 | 0.972  (0.753-1.256) | 0.896  (0.692-1.161) | 0.803  (0.577-1.120) | 0.717  (0.512-1.005) | 0.827  (0.561-1.220) | 0.677  (0.455-1.007) | 1.123  (0.795-1.586) | 0.967  (0.680-1.375) |
| 14 | 0.986  (0.763-1.273) | 0.794  (0.605-1.042) | 0.533**  (0.368-0.772) | 0.403***  (0.272-0.596) | 1.017  (0.700-1.477) | 0.614*  (0.410-0.919) | 1.343  (0.960-1.879) | 0.886  (0.617-1.271) |
| 15 | 0.955  (0.720-1.268) | 0.721*  (0.531-0.979) | 0.733  (0.502-1.070) | 0.515**  (0.342-0.777) | 1.005  (0.667-1.516) | 0.506**  (0.322-0.795) | 1.216  (0.838-1.766) | 0.692  (0.460-1.041) |
| Female | 0.896  (0.738-1.088) | 0.840  (0.691-1.022) | 0.513***  (0.391-0.672) | 0.472***  (0.359-0.622) | 0.316***  (0.232-0.431) | 0.271***  (0.197-0.371) | 0.387***  (0.298-0.502) | 0.339***  (0.259-0.443) |
| Parental education |  |  |  |  |  |  |  |  |
| High School Degree | 1.182  (0.833-1.677) | 1.192  (0.839-1.694) | 0.835  (0.515-1.356) | 0.844  (0.519-1.370) | 1.811*  (1.043-3.147) | 1.839*  (1.055-3.207) | 0.935  (0.619-1.414) | 0.951  (0.626-1.445) |
| Some College | 1.389  (0.965-2.000) | 1.372  (0.952-1.977 | 1.398  (0.864-2.262) | 1.378  (0.852-2.231) | 1.722  (0.962-3.082) | 1.650  (0.920-2.959) | 1.526  (1.000-2.327) | 1.524  (0.993-2.339) |
| 4 years of College or more | 1.617*  (1.108-2.359) | 1.657**  (1.135-2.419) | 1.165  (0.699-1.943) | 1.201  (0.721-2.002) | 1.755  (0.960-3.209) | 1.824  (0.996-3.339) | 1.686*  (1.078-2.639) | 1.785*  (1.134-2.810) |
| Race/Ethnicity |  |  |  |  |  |  |  |  |
| Black or African American | 0.818  (0.631-1.061) | 0.790  (0.609-1.026) | 1.225  (0.861-1.743) | 1.163  (0.816-1.656) | 0.966  (0.667-1.399) | 0.873  (0.602-1.267) | 0.644**  (0.464-0.893) | 0.619**  (0.444-0.863) |
| American Indian, Eskimo, Aleut | 1.015  (0.378-2.720) | 0.961  (0.356-2.592) | 0.488  (0.064-3.711) | 0.447  (0.058-3.429) | 0.000  (0.000-0.000) | 0.000  (0.000-0.000) | 0.619  (0.143-2.685) | 0.667  (0.153-2.907) |
| Asian or Pacific Islander | 0.522  (0.220-1.243) | 0.527  (0.222-1.255) | 0.903  (0.347-2.355) | 0.888  (0.340-2.321) | 0.812  (0.281-2.343) | 0.822  (0.281-2.409) | 0.259  (0.062-1.091) | 0.282  (0.067-1.190) |
| Other | 1.994*  (1.064-3.737) | 2.037*  (1.084-3.828) | 0.594  (0.140-2.521) | 0.601  (0.141-2.558) | 1.367  (0.471-3.967) | 1.485  (0.505-4.362) | 0.903  (0.311-2.623) | 0.903  (0.311-2.624) |
| Hispanic or Latino | 0.898  (0.671-1.202) | 0.872  (0.650-1.168) | 1.165  (0.785-1.730) | 1.114  (0.750-1.654) | 0.825  (0.526-1.293) | 0.798  (0.508-1.254) | 0.850  (0.597-1.211) | 0.853  (0.597-1.219) |
| Parental Health |  |  |  |  |  |  |  |  |
| Fair-Poor | 1.213  (0.899-1.637) | 1.201  (0.889-1.622) | 1.145  (0.750-1.749) | 1.128  (0.737-1.726) | 0.906  (0.562-1.461) | 0.894  (0.553-1.447) | 1.188  (0.822-1.718) | 1.216  (0.840-1.761) |
| No parent info | 0.972  (0.653-1.449) | 0.959  (0.643-1.431) | 1.040  (0.602-1.794) | 1.034  (0.598-1.786) | 0.984  (0.551-1.757) | 0.957  (0.532-1.722) | 1.319  (0.837-2.080) | 1.225  (0.765-1.961) |
| Employment Status |  |  |  |  |  |  |  |  |
| Part-time | 1.190  (0.974-1.454) | 1.150  (0.940-1.407) | 1.337*  (1.015-1.761) | 1.285  (0.974-1.696) | 1.151  (0.853-1.552) | 1.058  (0.782-1.432) | 0.886  (0.676-1.160) | 0.885  (0.674-1.163) |
| Full-time | 1.499  (0.894-2.512) | 1.433  (0.853-2.407) | 2.479**  (1.348-4.558) | 2.370**  (1.286-4.369) | 1.515  (0.721-3.088) | 1.384  (0.664-2.883) | 1.122  (0.578-2.178) | 1.225  (0.577-2.178) |
|  |  |  |  |  |  |  |  |  |
| Constant | 0.085***  (0.054-0.135) | 0.076***  (0.048-0.122) | 0.044***  (0.024-0.083) | 0.038***  (0.020-0.071) | 0.046***  (0.023-0.093) | 0.035***  (0.017-0.072) | 0.124***  (0.072-0.215) | 0.127***  (0.073-0.222) |

**p* < .05; ***p*<.01; ****p*<.001 Note: Reference outcome category is Abstainers.
