# Supplementary material for: Cognitive aptitude, peers, and trajectories of marijuana use from adolescence through young adulthood
Source: PLoS One. 2019 Oct 25;14(10):e0223152. doi: 10.1371/journal.pone.0223152 (PMC6814275; doi:10.1371/journal.pone.0223152)
Supplement: Attachment — Submitted filename: Response to Reviews -- PLoS One.docx [file pone.0223152.s002.docx]

Response to PLoS One Reviews -- PONE-D-19-16918

Thank you for the opportunity to revise this manuscript. We have provided our responses to the comments in blue text below. Within the tracked version of the revised manuscript, we have noted these changes in red text.

Editor Comments

1. Please ensure that you refer to Figures Appendix A in your text as, if accepted, production will need this reference to link the reader to the figure.

We have gone through the manuscript to ensure the references to the figure specify Appendix A as requested.

2. Please upload a copy of Figures 1 & 2, to which you refer in your text on page xx. If the figure is no longer to be included as part of the submission please remove all reference to it within the text.

This was in reference to the two panels of the figure in Appendix A. We have revised to simply state Appendix A for clarity.

3. Please include a separate caption for each figure in your manuscript.

There is but one figure. Appendix A is a single figure with the five trajectories spread across the two pages of the document for the purpose of legibility in review.

Reviewer #1: The focus of this manuscript is the potential impact of cognitive aptitude during adolescence on trajectories of marijuana use through age 26. The manuscript addresses a timely health behavior, presents a clear argument, and is well-written. While there are many strengths, there are also some major and minor concerns.

Major

The dependent variable for the analyses presented in this manuscript are latent classes of marijuana use based on trajectories over a ten-year period. However, all independent variables are from the baseline data. While this is less of a concern for the more variables more likely to be fixed (e.g., basic demographics), it is reasonable to expect that peer groups from age 16 to 26 would be very dynamic. Was peer use assessed at the multiple data collection time points? Can it be examined as a time-varying predictor? Similar comment for depression.

The time-varying nature of peer relationships is a consideration we have recognized. Unfortunately, these measures on peer groups were not collected annually within the NLSY and remain unavailable for analysis. The baseline measure of peer group deviance is the primary measure available. We note the inability to account for the time-varying nature of peer relationships in the revised manuscript. Similarly, the measure of depression (included in our models as a control variable prior to the marijuana use trajectory) was not an annually collected measure within the NLSY dataset. We also note this limitation in the revised manuscript.

The most recent year data was collected is 2011 – almost 9 years old. What evidence suggests that the findings from this cohort would generalize to the current generation of adolescents transitioning into adulthood?

There are unfortunately few national studies with panel designs and annually repeated measures of marijuana use that track transitions from adolescence to young adulthood, which requires an extended period of observation. There will inherently be a lag for any study requiring extended periods of observation, but as these data with the most recent data point utilized are only 8 years old (and only 6 years old since release for analysis), we remain confident that there are no more recent datasets with annual panel designs available to track these changes from adolescents to adulthood.

Minor

Page 16, Line 365. The text states the “analyses below” in reference to “Table 2”. You may want to consider referencing “Table 3” specifically rather than “below” for clarity.

We have made the recommended change on page 16.

Page 21, Line 469. Given that higher cognitive aptitude was associated with consistent use, it would be better to use “heavier” rather than “long-term”.

Per the reviewer’s request, we have revised the text on page 21 by referring to “extended heavy marijuana use” to denote both the duration and heavy frequency.

Please include sample sizes and referent groups for all tables.

We include sample sizes and referent groups within all of the tables.
